# ﻿*Cincticostellaebura* sp. nov., a new species of mayfly (Ephemeroptera, Ephemerellidae) from Thailand

**DOI:** 10.3897/zookeys.1130.91039

**Published:** 2022-11-21

**Authors:** Chonlakran Auychinda, Michel Sartori, Boonsatien Boonsoong

**Affiliations:** 1 Department of Biology, Faculty of Science, Silpakorn University, Nakhon Pathom, Nakhon Pathom Province, 73000, Thailand Silpakorn University Nakhon Pathom Thailand; 2 Museum of Zoology, Palais de Rumine, Place Riponne 6, CH-1005 Lausanne, Switzerland Museum of Zoology Lausanne Switzerland; 3 Department of Ecology and Evolution, Lausanne University, CH-101 Lausanne, Switzerland Lausanne University Lausanne Switzerland; 4 Animal Systematics and Ecology Specialty Research Unit (ASESRU), Department of Zoology, Faculty of Science, Kasetsart University, Bangkok 10900, Thailand Kasetsart University Bangkok Thailand; 5 Biodiversity Center Kasetsart University (BDCKU), Bangkok 10900, Thailand Biodiversity Center Kasetsart University Bangkok Thailand

**Keywords:** COI, ephemerellid mayfly, *insolta* complex, integrative taxonomy, *nigra* complex

## Abstract

A new species of ephemerellid mayfly, *Cincticostellaebura***sp. nov.**, is described based on larvae collected in a stream from Nan Province, Thailand. This new species is classified in the *nigra* complex of the genus *Cincticostella* based on morphological and COI phylogeny evidence. The new species is closely related to *C.nigra* (Uéno, 1928) and *C.funki* Martynov, Selvakumar, Palatov & Vasanth, 2021 based on body colour pattern. Investigation of the chorionic structure of the new species showed similar details to those of other species within this species complex. The phylogeny also placed this species into a monophyletic group with *C.nigra* (Uéno, 1928), *C.elongatula* (McLachlan, 1875) and *C.fusca* Kang & Yang, 1995.

## ﻿Introduction

The genus *Cincticostella* was established by Allen (1971) as a subgenus of *Ephemerella* Walsh, 1862, and was subsequently raised to generic level (Allen 1980). *Cincticostella* species are distributed in the eastern Palearctic and Oriental regions. The larvae are characterised by 1) anterolateral projections of the pronotum and mesonotum rounded and flat (contrary to sharp and acute projections in *Ephacerella*), and 2) a widened and flattened maxillary canine with reticulated ventral margin (Kluge 2004) or reduced to a denticulate blade (Jacobus and McCafferty 2008). Within the genus, some characters were used to distinguish and classify the different species into several species groups. For example, Allen (1975) divided the genus into two species complexes consisting of *insolta* and *nigra* complexes. These two species complexes are differentiated by a head with a pair of tubercles and expansion of the mid and hind femora with chalazae in the *insolta* complex, characters which are absent in the *nigra* complex. Allen (1980) proposed the subgenus Rhionella to accommodate the *insolta* complex, but this was refuted by Jacobus and McCafferty (2008) based on their phylogenetic reconstruction.

Recently, Martynov et al. (2021) proposed a gosei complex, separated from the *nigra* complex by several characters, such as segments I and II of the labial palp relatively narrow and elongated, moderate anterolateral emargination of the labrum and especially, the maxillary palp absent. Therefore, three species complexes are currently considered: the *nigra*, *insolta* and *gosei* complexes.

Currently, 22 species are recognised in the world, of which 17 are found in the Oriental region (Xie et al. 2009; Martynov et al. 2019; Auychinda et al. 2020a; Martynov et al. 2021). According to Martynov et al. (2021) the *insolta* complex comprises eight species, the *nigra* complex 13 species, and the *gosei* complex a single species. The genus *Cincticostella* has the highest diversity of the family Ephemerellidae in the Oriental region, but only three species are currently known in Thailand: *C.femorata* (Tshernova, 1972) and *C.insolta* (Allen, 1971) that belong to the *insolta* complex and *C.gosei* (Allen, 1975) from the *gosei* complex (Martynov et al. 2021).

In 2019, we collected larval material from Nan Province, Thailand. These specimens were morphologically identified and were found to share many characters with the East Palearctic species, *C.nigra* (Uéno, 1928) and Oriental species, *C.funki* Martynov, Selvakumar, Palatov & Vasanth, 2021. However, some characters were different and together with the distinct geographic or ecological distribution, we therefore classified these specimens as a new species and the first recorded species of the *nigra* complex in Thailand. The morphological characters of the mature larvae are described, including the chorionic structures, which were investigated by scanning electron microscopy (SEM). In addition, the COI gene of the new species was sequenced and a phylogenetic tree was reconstructed using our sequences and some *Cincticostella* COI sequences available in the GenBank database. Species delimitation was also based on the genetic distances using Kimura 2-parameter (K2P) analysis (Alexander et al. 2009; Tenchini et al. 2018).

## ﻿Materials and methods

### ﻿Specimen analysis

Larvae were collected using a D-frame kick net in the riffles of fast-flowing areas. The specimens were preserved in 95% ethanol and a whole larva was selected and dissected for morphological observation. The morphological characters were observed by permanent slide preparation using Euparal as a medium and observed by light microscopy. The eggs were also dissected from a late female larva. The chorionic structure was investigated by drying the eggs, coating them with gold, and observing them by SEM with a FEI Quanta 450. Final plates were prepared with Adobe Photoshop® CC 2020. Holotype and paratype specimens of the new species are deposited in the collections of the Zoological Museum at Kasetsart University in Bangkok, Thailand [**ZMKU**] and the Museum of Zoology in Lausanne, Switzerland [**MZL**].

### ﻿Molecular analysis

Thoracic muscles were dissected for DNA extraction. Total genomic DNA was extracted with a genomic DNA extraction kit (NucleoSpin, Macherey-Nagel, Germany) following the manufacturer’s protocol. The COI amplification was performed using LCO1490 and HCO2198 (Folmer et al. 1994). The polymerase chain reaction (PCR) conditions and procedure were performed as described previously (Auychinda et al. 2020c). Purification and sequencing were conducted by Macrogen, Inc. (South Korea). A Bayesian tree for ephemerellid mayflies was constructed for *Teloganopsis* spp., *Torleya* spp., available *Cincticostella* species (GenBank and BOLD system) and our sequences (658 bp) for the new species (MW633484), and one specimen of *C.insolta* (MW633483). We also added a specimen of *C.femorata* (Tshernova, 1972) from Chiang Mai Province, Thailand that was deposited in MZL (GBIFCH00763740_A01), and its COI was also extracted, sequenced, analysed and deposited in the GenBank database (MW633485). *Teloganellaumbrata* Ulmer, 1939 was used as an outgroup. The protocol for tree construction follows Auychinda et al. (2020c).

## ﻿Taxonomy

### ﻿Order Ephemeroptera

**Family Ephemerellidae Klapálek, 1909**

#### Genus *Cincticostella* Allen, 1971

##### ﻿ Cincticostella ebura sp. nov.

Taxon classificationAnimaliaEphemeropteraEphemerellidae

1929A9DE-1DE7-5630-AD3C-8BD1F5315C68

https://zoobank.org/99170F17-D407-4F9B-AF3C-72E18192A2A4

**Figure 1.**
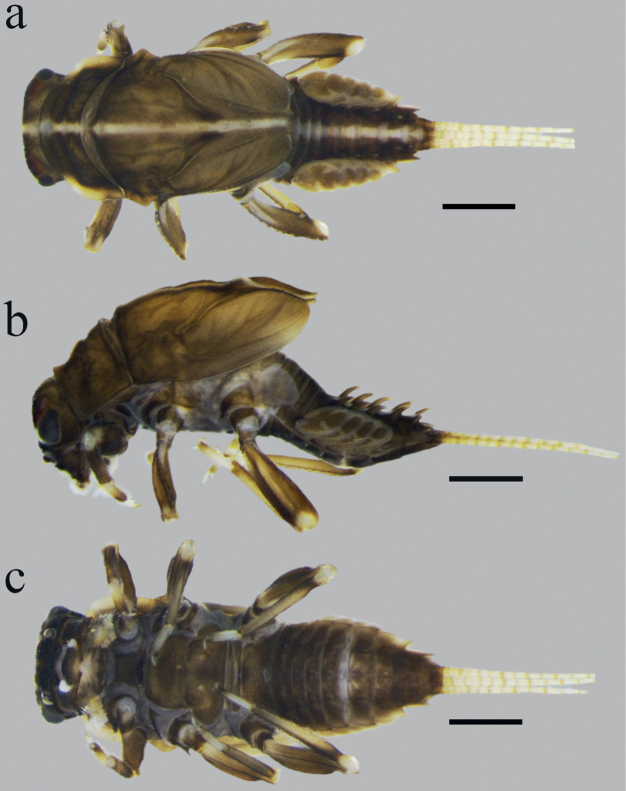
*Cincticostellaebura* sp. nov. **a** larval habitus in dorsal view **b** in lateral view and **c** in ventral view. Scale bars: 1 mm.

**Figure 2.**
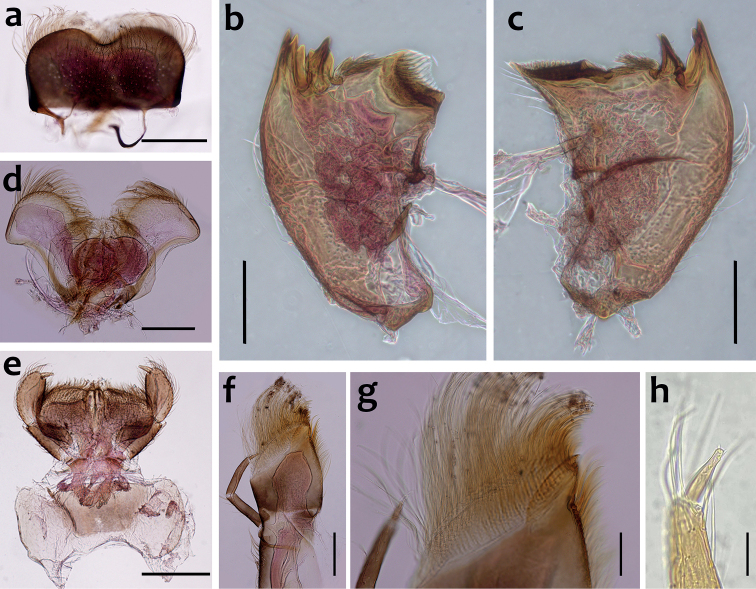
*Cincticostellaebura* sp. nov. **a** labrum **b** left mandible **c** right mandible **d** hypopharynx **e** labium **f** maxilla **g** galea-lacinia **h** segment III of maxillary palp. Scale bars: 0.2 mm (**a–f**); 0.05 mm (**g**); 0.035 mm (**h**).

**Figure 3.**
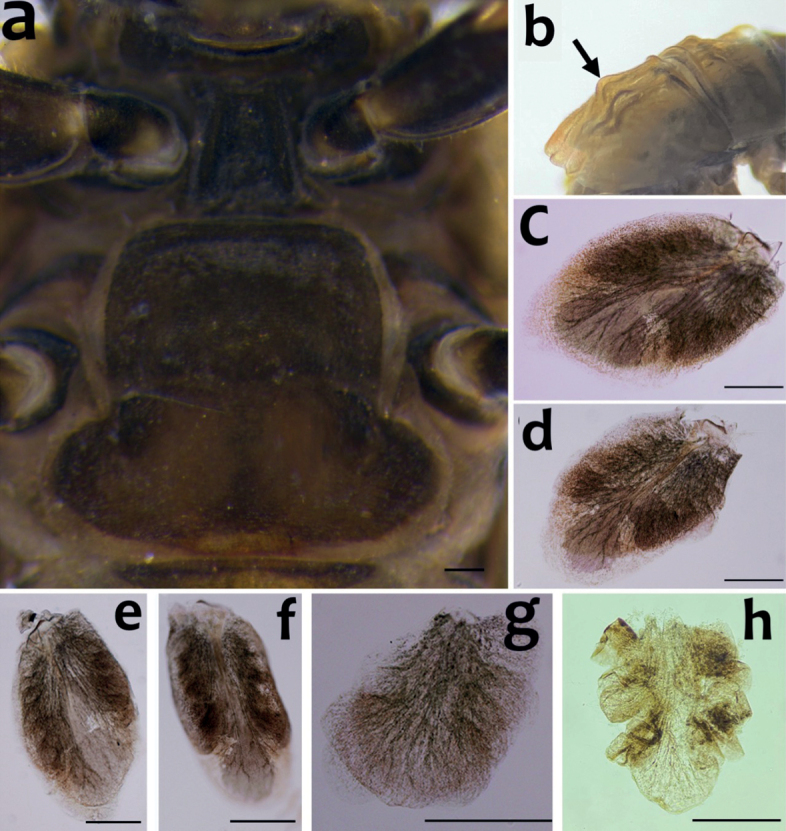
*Cincticostellaebura* sp. nov. **a** prosternum and mesosternum **b** pairs of tubercles (arrow) on mesothorax of early stage; **c–f** gills of segment III–VI **g** ventral lamella of gill of segment VII **h** ventral lamella of gill of segment VI. Scale bars: 0.2 mm.

**Figure 4.**
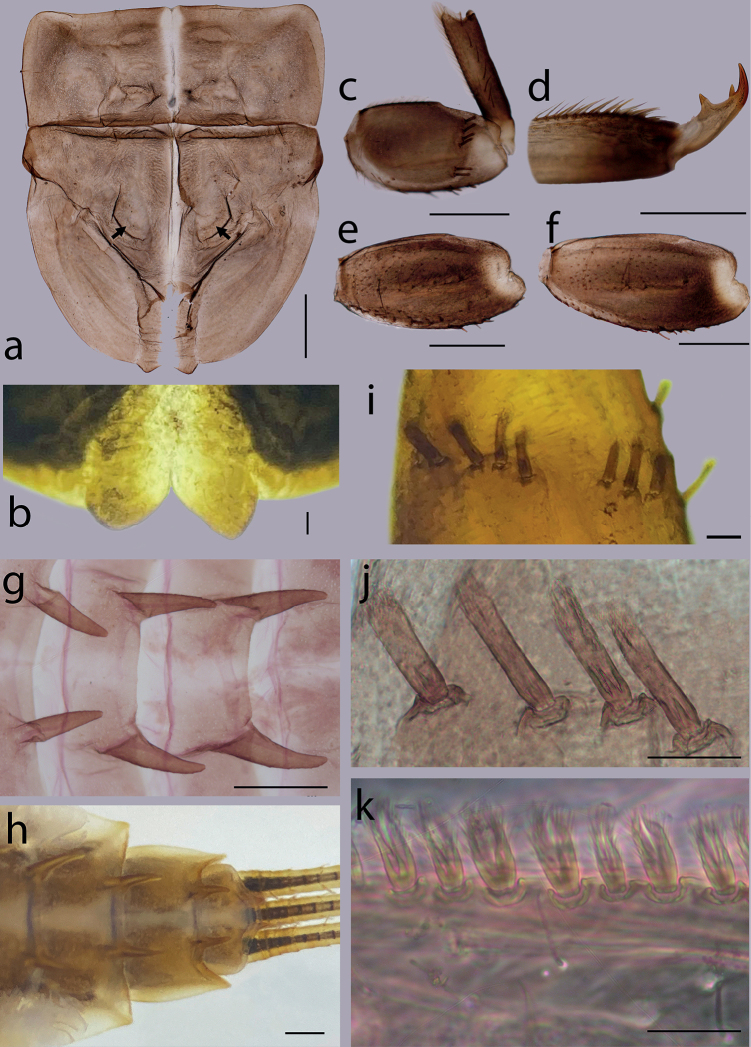
*Cincticostellaebura* sp. nov. **a** thorax in dorsal view, a pair of tubercles was indicated by arrows **b** posterior projection of mesonotum **c** foreleg **d** foretarsal claw **e** mid-femur **f** hind-femur **g** abdominal terga V–VII **h** abdominal terga VIII–X **i, j** setae on apically dorsal forefemoral surface **k** setae on posterior margin of abdominal terga. Scale bars: 0.2 mm (**a, b, c, e, f, g, h**); 0.05 mm (**d, j**); 0.01 mm (**k**).

**Figure 5.**
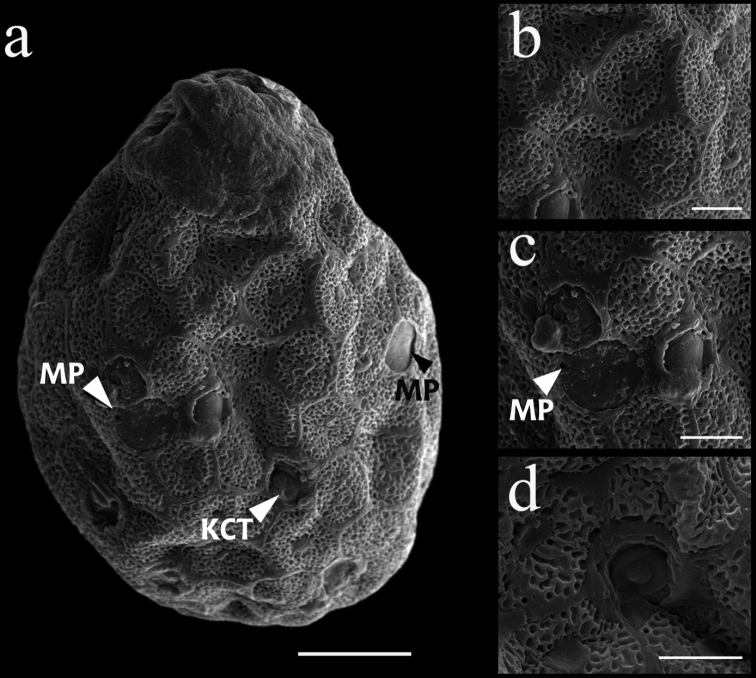
*Cincticostellaebura* sp. nov. **a** an overview of egg **b** chorionic surface **c** chorionic surface with micropyles **d** chorionic surface with KCT. Scale bars: 0.02 mm (**a**); 0.01 mm (**b–d**).

**Figure 6.**
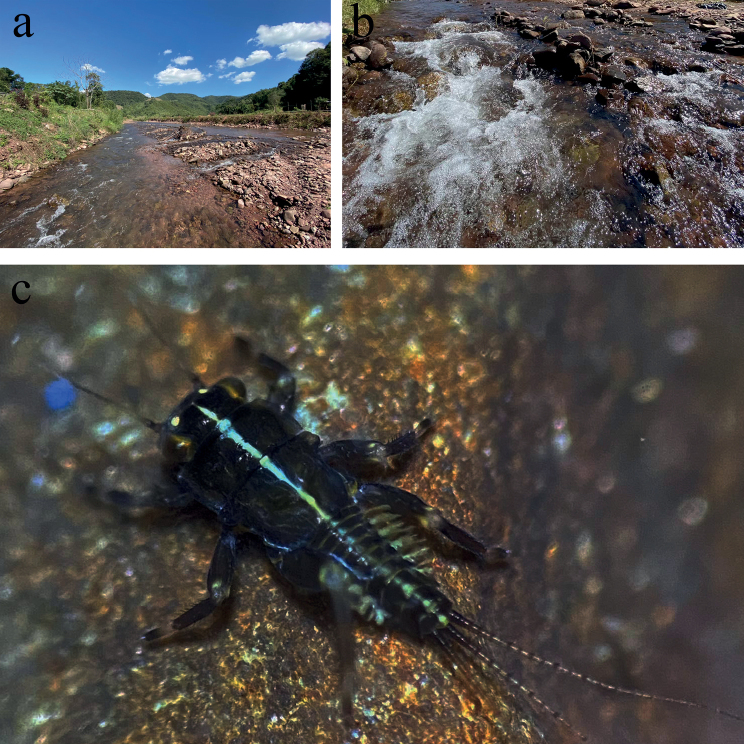
**a** The Mae Nam Wa stream, Bo Kluea district, Nan Province **b** microhabitat of the larvae of *Cincticostellaebura* sp. nov. **c***Cincticostellaebura* sp. nov. larva (living).

Figs 1 , 2 , 3 , 4 , 5 , 6c

###### Material examined.

***Holotype***: Male mature larva in ethanol, Thailand, Nan Province, Bo Kluea District, Mae Nam Wa stream, 19°16'22.6"N, 101°10'48.2"E, 848 m, 26.XI.2019, C. Auychinda leg. [**ZMKU**]. ***Paratypes***: 30 larvae in ethanol, one on slide, same data as holotype [**ZMKU**]; 4 larvae in ethanol, same data as holotype [**MZL** GBIFCH00977588].

###### Description.

**Mature larva** (in alcohol, Fig. 1; living, Fig. 6c). Body length (without cerci) 5.5–6.0 mm; cerci 6.0–8.5 mm; body brownish-black with a conspicuous dorsal median pale line from the head to tergum X (Figs 1a, 6c).

***Head*.** Black without tubercles, prominent bright ocelli; antennae three times longer than head length. Labrum densely covered with long fine setae, apicolateral angles rounded; apicomedially with deep emargination; ratio of emargination length to maximum labrum length = 1: 4.7 (Fig. 2a). Mandibles stout with numerous, hair-like setae on 2/3 proximal of dorsal and lateral surfaces (Fig. 2b, c). Left mandible: outer incisor composed of three acute teeth; inner incisor with one main stout and one inner vestigial tooth; prostheca with a bunch of hair-like setae on the inner side (Fig. 2b). Right mandible: outer incisor composed of two pointed teeth; inner incisor composed of two apically pointed teeth, orientated perpendicularly to the outer incisor; prostheca consisting of numerous hair-like setae (Fig. 2c) Hypopharynx: lingual surface covered with short setae, most abundant in apical part; superlinguae with numerous hair-like setae, apices rounded, posterolateral part concave (Fig. 2d). Labium with narrow elliptical glossae, almost four times longer than broad and covered with numerous short fine setae; paraglossae broad, semicircular, with surfaces covered with numerous simple setae. Labial palp three-segmented; segments I and II stout and equal in length, outer margin covered with hair-like setae, segment III spine-like in shape, 2.5 times longer than broad at the base (Fig. 2e). Maxillae slender; maxillary palpi long (0.46 mm), covered with tiny setae and three-segmented, length ratio from basal to apical segments = 4: 4: 1 (Fig. 2f), apex of segment II with long hair-like setae, segment III cone-shaped and with tiny short setae apically (Fig. 2h); apex of maxilla widened, surface with numerous long, hair-like setae; maxillary canine reduced to a small denticulated blade and less than half as long as crown, inner margin of galea-lacinia with 3–4 rows of simple setae (Fig. 2f, g).

***Thorax*.** Black with distinct white median line. Pronotum rectangular without clear anterolateral projections. Mesonotum with rounded anterolateral projections, outer margins not notched (Fig. 1a); mounted on slide, this character looks more angular (Fig. 4a); a pair of sub-median tubercles in the middle, a single posterior prominent median tubercle (Fig. 1b), posteriorly between fore wing pads with a pair of well-developed projections, angular with deep cleft (Figs 1a, 4a–b). Prothoracic sternum trapezoidal, mesothoracic basisternum rectangular, mesothoracic furcastemum broader than basisternum, oval transversely (Fig. 3a). Forefemora moderately dilated, ventral margin with fine setae, dorsal margin with spatulate setae most abundant in distal part, distal part of the dorsal surface with a transversal discontinuous row of 6–8 spatulate setae perpendicular to the femur (Fig. 4 c, i, j). Midfemora moderately expanded, dorsal margin smooth and with a row of short stout setae abundant in distal part (Fig. 4e). Hind femora moderately expanded, longer than mid femora, dorsal margin smooth, with a row of short stout setae from median to distal part (Fig. 4f). All claws similar, strongly hooked without apical setae, each with an acute basal and subapical tooth (Fig. 4d).

***Abdomen*.** Terga I–X each with a pair of posteromedian projections, well developed into strong tubercles of terga IV–VIII (Figs 1b, 4g); posterolateral projections of tergum VIII less developed (Fig. 4h); posterior margins of each tergum with bifurcate stout setae (Fig. 4k). Gills present on segments III–VII (Fig. 3c–h), all gills consistent with the diagnostic character of the genus *Cincticostella*: gill III without medial transverse band of weakened membrane; ventral lamella of gills III–V bifurcated (Fig. 3c–e), gill VI–VII non-bifurcate with marginal processes (Fig. 3f–h). Caudal filaments with whorls of dense setae on each segment.

***Eggs*.** Dissected from mature larva (Fig. 5). Ovoid, length ca 125 µm, width ca 110 µm; one pole covered with a dome-shaped polar cap, chorionic surface reticulated, almost hexagonal in formation, with a central spot (Fig. 5a, b). Equator with 4–6 micropyles, sperm guide circular and smooth (Fig. 5c). Rounded knob terminated coiled threads (KCT) especially abundant at the part opposite the polar cap (Fig. 5d).

**Adults.** Unknown.

###### Remarks.

The pair of sub-median tubercles in the middle of mesonotum of early stages is prominent and variable in number, 2 or 4 tubercles (Fig. 3b) which is similar to other Ephemerellidae such as *Notacanthellacommodema* (Allen, 1971) in which the tubercle numbers reduce and are more flattened in later stages (Auychinda et al. 2020b). On the contrary, the posterior median tubercle is distinct in all larval stages of the new species. Although, *C.funki* has no distinct prominent tubercle on their posterior median mesothorax in later stages, this tubercle is distinct in the small larval stages (A. Martynov, pers. comm.)

###### Diagnosis.

The larva of *Cincticostellaebura* sp. nov. has a well-marked white median line along its body that can be used to separate it from other *Cincticostella* species. However, this pattern is also present in *C.nigra* (Uéno, 1928) and *C.funki* Martynov, Selvakumar, Palatov & Vasanth, 2021, and the body shape is quite similar (Uéno 1928; Ishiwata 2003; Martynov et al. 2021). Although, claws of *C.ebura* sp. nov. and *C.funki* are hooked with an acute basal and subapical tooth, this character is absent in *C.nigra*, where a row of 6–8 teeth of unequal size can be found (Uéno 1928, fig. 9h–i) or 5–8 denticles of tarsal claws (Ishiwata 2003). In addition, the dorsal surface of the mid- and hind femora of *C.ebura* sp. nov. possess clavate setae while in *C.nigra*, these setae are absent (Ishiwata 2003, figs 48, 52). Furthermore, *C.ebura* sp. nov. can be distinguished from *C.nigra* and *C.funki* based on the combination of following characteristics: 1) small denticulate blade maxillary canine; 2) maxillary palp segment III cone-shaped; 3) all abdominal terga with long pairs of tubercles, especially on terga IV to VIII, on tergum X small and pointed; 4) anterolateral projection of the pronotum absent; 5) mature larvae length is almost less than two times of *C.funki*; 6) mesonotum with single prominent median posterior tubercle and posteriorly with a pair of well-developed angular projections; and 7) a transverse discontinuous row of stout setae and without extra setae on surface of forefemora.

###### Etymology.

The specific epithet ‘ebura’, which means ivory, is a reference to the pairs of long and curve tubercles on the abdominal posteromedian margins.

###### Habitat and ecology.

The type locality of *Cincticostellaebura* sp. nov. is the Mae Nam Wa stream, Nan Province, Thailand (Fig. 6a). The larvae were collected by handpicking and D-frame net kicking methods from cobble and pebbles within moderate- to fast-flowing current of run/riffle areas (Fig. 6b). This study site also shows a high taxa richness of Ephemerelloidea larvae, as other species, including *C.insolta* (Allen, 1971), *Notacanthellaquadrata* (Kluge & Zhou, 2004), *N.commodema* (Allen, 1971), *Dudgeodes* sp. and *Vietnamellananensis* Auychinda, Sartori & Boonsoong, 2020, co-occurred with the larvae of *C.ebura* sp. nov.

###### Distribution.

Nan Province, northern Thailand.

### ﻿Molecular analysis

**Figure 7.**
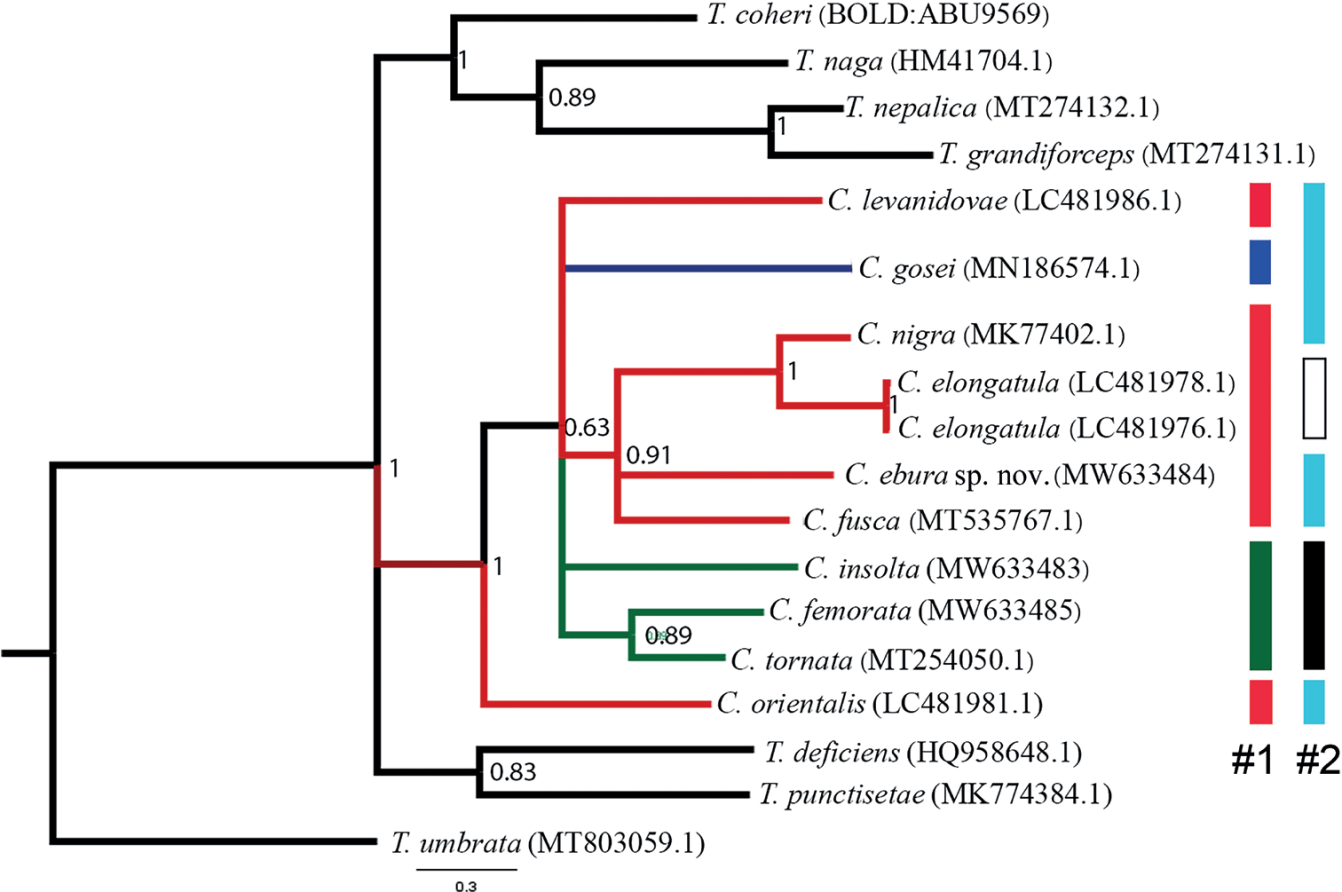
Bayesian inference of COI sequences of some ephemerellid mayflies including *Teloganopsis*, *Torleya* and *Cincticostella* with probability branch support and GenBank accession numbers, or BOLD numbers, in brackets. The color bars indicate the species complex of the genus *Cincticostella*. The first column (#1) is the species complex following Martynov et al. (2021): red = *nigra* complex, blue = *gosei* complex and green = *insolta* complex. The second column (#2) follows Kluge (2021): sky blue = *Cincticostella*/g4, black = *Rhionella* and blank box = uncertain placement (*Ephemerella*/fg3 INCERTAE SEDIS). *Teloganellaumbrata* (Ephemerelloidea; Teloganellidae) was chosen as an outgroup.

The Bayesian phylogenetic tree reconstruction of COI showed that *Cincticostella* forms a monophyletic lineage which is distinctly separated from the other ephemerellid mayflies, with high probability branch support (Fig. 7). Our reconstruction contained ten species of *Cincticostella*, and the interspecific genetic distances ranged from 15–26%. *Cincticostellaebura* sp. nov. differed from other species by a range of 21 to 26% (Table 1).

**Table 1.** Pairwise genetic distances (COI) between species of *Cincticostella* using the Kimura 2-parameter.

Species	K2P genetic distances								
	1	2	3	4	5	6	7	8	9
1. *C.ebura* sp. nov.									
2. *C.nigra*	0.22								
3. *C.elongatula*	0.24	0.15							
4. *C.levanidovae*	0.24	0.24	0.24						
5. *C.tornata*	0.23	0.25	0.25	0.26					
6. *C.femorata*	0.23	0.24	0.24	0.20	0.16				
7. *C.gosei*	0.23	0.21	0.25	0.23	0.22	0.23			
8. *C.insolta*	0.22	0.25	0.23	0.22	0.22	0.22	0.22		
9. *C.orientalis*	0.26	0.26	0.26	0.25	0.22	0.25	0.23	0.23	
10. *C.fusca*	0.21	0.22	0.24	0.23	0.22	0.24	0.23	0.24	0.23

## ﻿Discussion

Our morphological evaluation of *C.ebura* sp. nov, especially body coloration, revealed some similarities with *C.nigra* and *C.funki*. However, these three species inhabit different geographic areas, as *C.nigra* is only reported from the East Palaearctic (Uéno 1928; Ishiwata 2003), while *C.funki* and *C.ebura* sp. nov. both have an Oriental distribution in northern India and northern Thailand, respectively. In addition, ecological factors are also different between the habitats of *C.ebura* sp. nov. and *C.funki*. The larvae of *C.ebura* sp. nov. were collected from a stream which temperature was 18–20 °C in sampling period at 848 m a.s.l. *Cincticostellafunki* inhabits in lower water temperature and higher altitude, 12 °C in the sampling period and 1285 m a.s.l. (Martynov et al. 2021).

The egg chorionic structure shows a similar pattern to that of the other *Cincticostella* species, including *C.levanidovae*, *C.elongatula*, *C.nigra*, *C.fusca*, *C.orientalis*, *C.colossa* and *C.femorata* (Kang and Yang 1995; Ishiwata 2003; Jacobus and McCafferty 2008; Zheng and Zhou 2021). It has hexagonal ridges with marks at the centre; the marks vary both in shape and in number therefore can be used to identify species complex of this genus. The dichotomous key to species using chorionic structure is presented below. However, *C.ebura* sp. nov. cannot be separated from *C.colossa*, *C.fusca* and *C.orientalis* by the shape and number of the marks. The egg size can be helpful because *C.ebura* sp. nov. has the smallest egg compared to the others.

From our results, *C.ebura* sp. nov. belongs to the *nigra* complex according to Martynov et al. (2021), or *Cincticostella*/g4 sensu Kluge (2021) based on morphological and molecular evidence. Although this genus has a high number of species, only four of them are found in Thailand, *C.ebura* sp. nov. being the first species from the *nigra* complex to be reported from Thailand.

Our molecular results support the placement of *C.ebura* sp. nov. into the *nigra* complex. In addition, our analysis supports the placement of *C.elongatula* (McLachlan, 1875) by Martynov et al. (2021) into the *nigra* complex. Our tree topology displays several polytomies and did not show the species complexes proposed by both Kluge (2021) and Martynov et al. (2021). However, our reconstruction (Fig. 7) seems to indicate that the *insolta* complex may well be a monophyletic lineage corresponding to the subgenus Rhionella.

In our reconstruction, *C.orientalis* (Tshernova, 1952) was recovered as the sister clade of all *Cincticostella* species, whereas Martynov et al. (2021) include it in the *nigra* complex. Our results also support *C.orientalis* as a valid species and not a synonym of *C.levanidovae* (Tshernova, 1952) as proposed by Tshernova et al. (1986) and by Kluge (2021). The species complexes relationship may be solved when more molecular data, both nuclear and mitochondrial DNA, becomes available (Ogden et al. 2019).

### ﻿Key to the mature nymphs of *Cincticostella* species in Thailand

**Table:** 

1	Mid- and hind femora expanded; head with a pair of tubercles	**2**
–	Mid- and hind femora not expanded; head without tubercles	**3**
2	Pronotum with broad and extended anterolateral projection around head capsule	***C.femorata***
–	Pronotum with moderately anterolateral projection…	***C.insolta***
3	Body black without median pale line; maxillary without palpi	***C.gosei***
–	Body black with median pale line along the body; maxillary with three-segmented palpi	***C.ebura* sp. nov.**

### ﻿Key to known egg structures of *Cincticostella* species (excluding *C.gosei*)

**Table:** 

1	Chorion covered with broken reticulation (Ishiwata, 2003, figs 7, 8)	***C.levanidovae***
–	Chorion covered with not broken reticulation	**2**
2	Chorionic surface with one tubercle (rarely two) at the centre of hexagonal ridge	**3**
–	Chorionic surface with a variety of tubercles (1–5) at the centre of hexagonal ridge (Kang and Yang 1995, figs 11, 12, 14, 15; Ishiwata 2003, figs 15, 16)	***C.colossa* , *C.fusca* , *C.orientalis* and *C.ebura* sp. nov.**
3	Egg relatively large, surface seems to be rough (length 162–168 µm, width 116–120 µm) (Ishiwata 2003, figs 3, 4, 11, 12)	***C.elongatula* and *C.nigra***
–	Egg relatively small, surface seems to be smooth (length 152 µm, width 114.6 µm) (Zheng and Zhou 2021, fig. 8)	***C.femorata***

## Supplementary Material

XML Treatment for Cincticostella ebura
